# The Influence of Homologous Arm Length on Homologous Recombination Gene Editing Efficiency Mediated by SSB/CRISPR-Cas9 in *Escherichia coli*

**DOI:** 10.3390/microorganisms12061102

**Published:** 2024-05-29

**Authors:** Ran Chai, Jiaxiang Guo, Yue Geng, Shuai Huang, Haifeng Wang, Xinding Yao, Tao Li, Liyou Qiu

**Affiliations:** 1 School of Environmental Engineering, Yellow River Conservancy Technical Institute, Henan Engineering Technology Research Center of Green Coating Materials, Kaifeng 475004, China; chairan@yrcti.edu.cn (R.C.);; 2 College of Life Sciences, Henan Agricultural University, Key Laboratory of Enzyme Engineering of Agricultural Microbiology, Ministry of Agriculture and Rural Affairs, Zhengzhou 450046, China; 3 College of Applied Engineering, Henan University of Science and Technology, Sanmenxia 472000, China

**Keywords:** CRISPR-Cas9 genome editing, SSB/CRISPR-Cas9, homologous recombination, double-strand breaks, dsDNA repair, lengths of homologous hrms

## Abstract

The precise editing of genes mediated by CRISPR-Cas9 necessitates the application of donor DNA with appropriate lengths of homologous arms and fragment sizes. Our previous development, SSB/CRISPR-Cas9, has demonstrated high efficiency in homologous recombination and non-homologous end joining gene editing within bacteria. In this study, we optimized the lengths and sizes of homologous arms of the donor DNA within this system. Two sets of donor DNA constructs were generated: one set comprised donors with only 10–100 bp homologous arms, while the other set included donors with homologous arms ranging from 10–100 bp, between which was a tetracycline resistance expression cassette (1439 bp). These donor constructs were transformed into *Escherichia coli* MG1655 cells alongside pCas-SSB/pTargetF-*lacZ*. Notably, when the homologous arms ranged from 10 to 70 bp, the transformation efficiency of non-selectable donors was significantly higher than that of selectable donors. However, within the range of 10–100 bp homologous arm lengths, the homologous recombination rate of selectable donors was significantly higher than that of non-selectable donors, with the gap narrowing as the homologous arm length increased. For selectable donor DNA with homologous arm lengths of 10–60 bp, the homologous recombination rate increased linearly, reaching a plateau when the homologous arm length was between 60–100 bp. Conversely, for non-selectable donor DNA, the homologous recombination rate increased linearly with homologous arm lengths of 10–90 bp, plateauing at 90–100 bp. Editing two loci simultaneously with 100 bp homologous arms, whether selectable or non-selectable, showed no difference in transformation or homologous recombination rates. Editing three loci simultaneously with 100 bp non-selectable homologous arms resulted in a 45% homologous recombination rate. These results suggest that efficient homologous recombination gene editing mediated by SSB/CRISPR-Cas9 can be achieved using donor DNA with 90–100 bp non-selectable homologous arms or 60–100 bp selectable homologous arms.

## 1. Introduction

CRISPR/Cas9 is a widely used genetic editing tool in eukaryotic organisms [1,2] and consists of two main components: the Cas9 protein and the sgRNA. Within recipient cells, the Cas9 protein is guided by the sgRNA to specific target sites within the genome. Upon formation of a complex between the sgRNA molecule and the Cas9 protein, it arrives at the protospacer adjacent motif (PAM) and the target sequence. When the 20-nucleotide sequence at the 5′ end of the sgRNA matches the sequence at the target site, the Cas9 protein induces a double-strand break (DSB) approximately three base pairs upstream of the PAM. Subsequently, the cell repairs these DSBs through either non-homologous end joining (NHEJ) or homologous recombination (HR) [3,4]. However, due to the common lack of homologous recombination repair systems in bacteria and their limited capability for non-homologous end joining repair, the utilization of CRISPR gene editing in bacteria often results in low efficiency or even ineffectiveness [5,6].

The NHEJ pathway requires the involvement of two proteins, Ku and LigD. In prokaryotes, this pathway is relatively weak and is often insufficient for repairing double-strand breaks (DSBs), leading to extremely low repair efficiency [6]. In *Escherichia coli*, the lack of an NHEJ repair mechanism further exacerbates the challenge of DNA repair [7]. The NHEJ process is inherently stochastic, potentially resulting in small, random insertions or deletions within genes at the break sites, leading to frameshift mutations and subsequent loss of gene function [8,9]. Therefore, the NHEJ pathway cannot achieve precise editing of bases. HR repair allows for precise gene editing, including knock-ins, replacements, knockouts, and point mutations. To compensate for the lack of HR repair in prokaryotic cells, the use of the λ-Red recombination system to mediate HR has become an efficient method for bacterial genome editing [10,11,12]. The λ-Red recombination system consists of Exo (Redα), Beta (Redβ), and Gam (Redγ) proteins. Exo is a 5′-to-3′ double-stranded DNA-dependent exonuclease that generates single-stranded DNA intermediates. Beta is a single-stranded DNA binding protein that facilitates pairing or annealing between complementary single-stranded DNA molecules. Gam protects double-stranded DNA from degradation by inhibiting cellular nucleases, thereby enhancing recombination efficiency [13]. The efficiency of editing single gene loci in *Escherichia coli* using the pCas/pTargetF system ranges from 6% to 92%. This efficiency is largely dependent on the length of homologous arms and the size of the donor DNA fragment [12]. The pCas/pTargetF system has been employed in various bacteria species [14,15,16,17]. Our previous research utilized the SSB/CRISPR-Cas9 system that is constructed by replacing the λ-Red recombination system with the SSB protein gene. This system leverages the dual functionality of SSB in both HR and NHEJ repair processes [18], resulting in a broader application scope and higher gene editing efficiency [19].

When employing CRISPR for HR gene editing, much attention has been directed towards factors such as the transformation method [20,21], minimizing off-target effects [22,23,24], and optimizing sgRNA design [25]. While these are crucial factors influencing CRISPR in the process of HR repair for gene editing, the length of homologous arms, whether the homologous fragment carries a resistance gene expression cassette, the number of cleavage sites, and the number of genes to be edited simultaneously all significantly affect gene editing efficiency. These are commonly encountered issues in practical applications. However, there is currently a lack of research in these areas.

The HR mechanism requires a donor DNA template for precise DSB repair. When performing single base mutations, single-stranded DNA (ssDNA) is commonly used as the donor DNA fragment. During HR repair with ssDNA, only the Beta protein is involved, and its length typically ranges from 20 to 70 bp [26]. When inducing single base mutations in *Escherichia coli*, Jiang et al. utilized ssDNA with a length of 56 bp [27]. When performing complete gene knockout or gene insertion, double-stranded DNA (dsDNA) fragments are commonly preferred. The choice of fragment length varies considerably among different studies. For instance, in editing Bacillus subtilis, Liu et al. utilized the CRISPR/Cas9n system with homologous arms of 500 bp in length both upstream and downstream of the target region. They achieved deletions ranging from 1 to 8 kb and insertions ranging from 1 to 2 kb, with efficiencies of at least 80% and 90%, respectively [28]. Using the CRISPR/Cas9 system, Watzlawick et al. inserted the *ganA* gene into the chromosome with homologous arm lengths ranging from 700 to 800 bp [29]. Lim et al. knocked out six genes, including *aprE*, with each fragment containing 500 bp homologous arms upstream and downstream [30]. Price et al., when knocking out the *arpE* gene, employed homologous arms of 972 bp and 999 bp upstream and downstream, respectively, achieving a gene editing efficiency of 76% [31]. When editing genes in *Escherichia coli*, Ao et al. utilized the CRISPR/Cas12a system for gene knockout. They found that, with a 50 bp homologous arm length donor DNA provided by plasmid, no transformants were obtained. Under conditions with 100 bp homologous arms, the gene editing efficiency was approximately 30% [32]. When using CRISPR/Cas9 to knock out the *qseB* gene, Gou et al. employed upstream and downstream homologous fragments of 369 bp and 348 bp, respectively [33]. Abdelaal et al., for the production of butanol, inserted an optimized pathway cassette into the genome. They used 600 bp homologous arms upstream and downstream for gene editing [34]. Li et al. achieved a large fragment gene insertion of 15.4 kb into the genome using homologous arm segments of approximately 500 bp each [35]. Sun et al., utilizing the CT-CRISPR/Cas9 system for gene knockout, employed short homologous arms of 50 bp each upstream and downstream to achieve gene knockout. However, the knockout efficiency was only approximately 5.88% [36]. In our previous study, when editing *Escherichia coli* MG1655 strain using the SSB/CRISPR-Cas9 system, we utilized fragments with 200 bp homologous arms, resulting in a gene editing efficiency of approximately 89.1% [19].

To investigate the optimal length of homologous arms for gene knockout or insertion using the SSB/CRISPR-Cas9 gene editing system, we first conducted experiments using a series of gradient lengths of donor dsDNA fragments. The aim of this was to determine the shortest and most effective length of homologous arms, as well as to assess the impact of varying homologous arm lengths on gene editing efficiency. Furthermore, in the process of CRISPR gene editing, depending on the application scenario, homologous fragments may sometimes need to include selection markers (such as antibiotic resistance gene expression cassettes) for subsequent screening. It is important to explore whether the optimal length of homologous arms changes under such circumstances and whether it affects gene editing efficiency. Consequently, we also designed a series of homologous fragments containing selection markers for gene editing experiments, aiming to identify both the optimal length of homologous arms and the shortest length required to achieve successful gene editing. These studies are expected to provide essential references for bacterial homologous recombination editing.

When targeting a larger genomic region for knockout, whether introducing a single cleavage site within the target region to induce a DSB or introducing double cleavage sites near both ends of the target region results in different efficiencies in gene editing remains unexplored. Additionally, it is unclear whether double cleavage sites can enhance gene editing efficiency and whether the presence of selection markers affects gene editing. To address these questions, we conducted experiments setting up double cleavage sites and utilized two types of homologous fragments: one without selection markers and the other with selection markers. These experiments aimed to compare the outcomes with those obtained by introducing single cleavage sites, providing insights into the impact of cleavage site configuration and the presence of selection markers on gene editing efficiency.

In the construction of engineered strains, it is often necessary to edit multiple genes. However, traditional editing systems typically allow editing of only one gene at a time, with fewer instances where three genes can be simultaneously edited, and with lower efficiency. When editing *Escherichia coli* MG1655, Ao et al. utilized the CRISPR-Cas12a system and CRISPR-Cas9, each capable of editing up to three genes (*galK*, *lacZ*, and *pyrF*) simultaneously. The editing efficiency was approximately 20% [32]. Jiang et al. achieved an efficiency of approximately 47% when simultaneously editing three genes by constructing the gRNA expression cassette and donor DNA on a plasmid [12]. Feng et al. established the multiplex genome engineering (CMGE) technique with CRISPR-Cas9 assistance, employing multiple gRNA plasmids to simultaneously edit four genes. The efficiency was able to exceed 30%, with an impressive 88.3% efficiency observed for the simultaneous editing of three genes [37], However, these methods involved integrating the donor DNA into a vector rather than using linear DNA as the donor, which increases the complexity of application and operation. Li et al. opted to use linear ssDNA as the editing template, achieving an efficiency of 83% when simultaneously editing two genes. However, when editing three genes simultaneously, the editing efficiency dropped to approximately 23% [38]. Lim et al. constructed a plasmid that separated three sgRNAs with the ssDNA, while individually inserting four base pairs into each of the *galK*, *xylB*, and *srlD* genes. The editing efficiency was approximately 13% [39]. Jiang et al. employed linear dsDNA for gene knockout in *Streptococcus pneumoniae*, achieving an efficiency of 75% for editing two genes simultaneously [27]. To our knowledge, there have been no reports of using linear dsDNA as donor fragments for simultaneous editing of three genes. Addressing the question of whether our previously constructed SSB/CRISPR-Cas9 system could perform edits on multiple genes simultaneously, this study investigated a series of homologous fragments (linear dsDNA) of different lengths, both with and without selection markers, without the need for construction on vectors. The study explored the impact of different lengths of homologous arms on the editing efficiency of the SSB/CRISPR-Cas9 editing system, conducted single-point and double-point gene editing, simultaneously achieved homologous recombination editing of three genes, determined the optimal conditions for using the SSB/CRISPR-Cas9 editing system in bacteria, and investigated whether SSB/CRISPR-Cas9 could edit multiple genes simultaneously.

## 2. Materials and Methods

### 2.1. Bacterial Strains, Plasmids, and Culture Conditions

The bacterial strains and plasmids used in this study are listed in Table 1. Strains were grown in lysogenic broth (LB) medium (1% (*w*/*v*) tryptone, 0.5% (*w*/*v*) yeast extract, 1% (*w*/*v*) NaCl) at either 37 °C or 30 °C. Kanamycin (25 µg/mL), spectinomycin (50 µg/mL), or tetracycline (15 µg/mL) were added as necessary. *Escherichia coli* DH5α was utilized for plasmid construction and maintenance, while *Escherichia coli* MG1655 was employed for gene editing purposes.

### 2.2. Plasmid Construction

To construct the pTargetF-*lacZ*-2 and pTargetF-3 plasmids, DNA fragments respectively containing 2 and 6 N_20_ sequences (Figures S1 and S2) were synthesized by BGI (Shenzhen, China). The lengths of these DNA fragments were 364 bp and 1204 bp, respectively, and they included the DNA regions corresponding with the sgRNAs. Both DNA fragments contained identical BamHI and EcoRI restriction enzyme sites at their ends. Digestion reactions were performed using BamHI and EcoRI enzymes (Beverly, MA, USA) on the pTargetF plasmid and the synthesized DNA fragments, followed by ligation reactions using T4 DNA ligase (Beverly, MA, USA) to obtain the pTargetF-*lacZ*-2 and pTargetF-3 plasmids. The digestion and ligation procedures were conducted according to the manufacturer’s instructions. In brief, the plasmid and the fragments to be ligated were adjusted to a 1:1 molar ratio and subjected to digestion with the corresponding restriction enzymes at 37 °C for 2–6 h. After purification of the reaction products, they were mixed and incubated with T4 DNA ligase at 37 °C for 12 h. The resulting products were directly transformed into DH5α competent cells, cultured in LB medium for 12 h, and the plasmids were then extracted and sequenced for further use.

### 2.3. Construction of Donor DNA

All primers used in this experiment are listed in Table 2. Three types of donor DNA were used. The first and second types consisted of a series of donor DNA fragments of varying lengths for knocking out the *lacZ* gene in *Escherichia coli* MG1655. The first type of donor DNA fragments did not contain the tetracycline resistance gene cassette, while the second type of donor DNA fragments contained the selection marker (antibiotic resistance gene expression cassette) in the middle of the homologous arms. Both types of donor DNA fragments contained homologous arms flanking the target region (upstream and downstream), with gradients of 10 bp, 20 bp, 30 bp, 40 bp, 50 bp, 60 bp, 70 bp, 80 bp, 90 bp, and 100 bp, totaling 10 different lengths.

The donor DNA fragments of the first type of varying lengths were directly synthesized by BGI (Shenzhen, China) (Table S1). The donor DNA fragments of the second type of varying lengths were constructed by PCR, using primers containing different lengths of homologous arms in the target region. The primers used were pC01/pC02, pC03/pC04, pC05/pC06, pC07/pC08, pC09/pC10, pC11/pC12, pC13/pC14, pC15/pC16, pC17/pC18, and pC19/pC20. These were amplified using the synthesized tetracycline resistance gene cassette DNA fragment (Figure S3) as a template and then purified.

The third type of donor DNA contained three DNA fragments, jointly used for knocking out the *RecA*, *RecBCD*, and *SSB* genes in *Escherichia coli* MG1655. These three DNA fragments contained homologous arms of 100 bp in length flanking the target region (upstream and downstream) and were directly synthesized by BGI (Shenzhen, China) (Figure S4).

### 2.4. Genome Editing 

*Escherichia coli* MG1655 cells were co-transformed with pCas-SSB/pTargetF-*lacZ*, pCas-SSB/pTargetF-*lacZ*-2 and pCas-SSB/pTargetF-3 plasmids along with the corresponding donor DNA fragments using the standard CaCl_2_ transformation procedure [41]. Each plasmid (200 ng) and donor DNA fragment (400 ng) was transformed into 100 µL of competent cells suspension. After heat shock, the mixture was immediately added to 0.9 mL fresh LB medium supplemented with 10 mM arabinose, followed by incubation at 30 °C and 220 rpm for 2 h to allow cell recovery. Subsequently, the culture was divided into three equal aliquots and spread onto three LB plates supplemented with 1 mM IPTG, 40 μg/mL X-gal, and either 25 μg/mL kanamycin, 50 μg/mL spectinomycin, or 15 μg/mL tetracycline. The plates were then incubated overnight at 37 °C. The transformation experiment was repeated three times.

Transformants were identified by blue-white screening, colony PCR, and DNA sequencing. Primers pC21/pC22 were used to detect the white colonies that were obtained, and the same primers were used for sequencing of the obtained PCR products. Primers pC23/pC24, pC25/pC26, and pC27/pC28 were used to detect transformant colonies with *RecA*, *RecBCD*, and *SSB* genes knocked out, respectively, and the same primers were used for sequencing of the PCR products obtained.

Both CRISPR plasmids used in this study conferred kanamycin and spectinomycin resistance to their host bacteria. Therefore, transformation efficiency was calculated by counting the total number of colonies grown on LB plates supplemented with 40 μg/mL X-gal and either 25 μg/mL kanamycin, 50 μg/mL spectinomycin, or 15 μg/mL tetracycline. Gene editing efficiency was determined by calculating the ratio of white colonies to total colonies (white + blue colonies) grown on the plates. To determine the homologous recombination rate, 15 white colonies were randomly selected for lacZ gene region PCR amplification and sequencing (repeated three times), and the proportion of white colonies with the lacZ gene knocked out by homologous recombination was multiplied by the gene editing efficiency.

## 3. Results

### 3.1. Impact of Homologous Arm Length of Donor DNA on Transformation Efficiency

**Figure 1:**
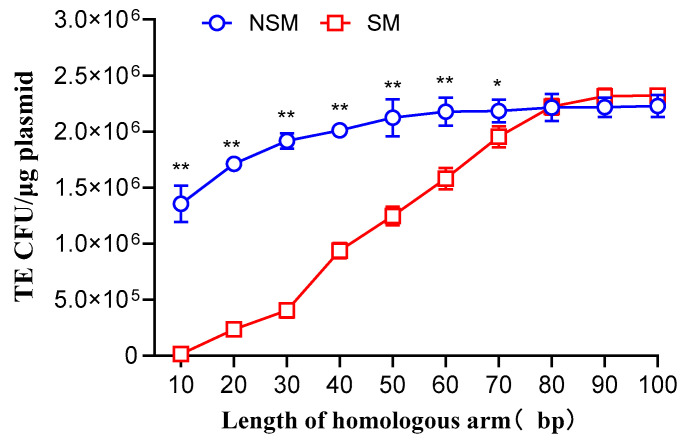
Transformation efficiency (TE) of donor DNA fragments with different lengths of homologous arms, with and without selection markers, co-transformed with pCas-SSB/pTargetF-*lacZ* into *Escherichia coli* MG1655. NSM, no selection marker; SM, selection marker. Data represent the mean of three independent experiments, with standard deviation (SD) as the error bars and are based on one-way ANOVA analysis. **, *p* < 0.001; *, *p* < 0.005.

Different lengths of homologous arm donor DNA fragments, both with and without selection markers, were co-transformed with pCas-SSB/pTargetF-*lacZ* into MG1655 cells (Figure S5). For donor DNA fragments without selection markers, the transformation efficiency increased linearly from 1.35 × 10^6^ CFU/μg plasmid at 10 bp to 2.12 × 10^6^ CFU/μg plasmid at 50 bp (*p* < 0.001). When the homologous arm length was between 50–100 bp, the transformation efficiency reached a plateau, with an average of 2.19 × 10^6^ CFU/μg plasmid. For donor DNA fragments with selection markers, the transformation efficiency increased linearly from 1.33 × 10^4^ CFU/μg plasmid at 10 bp to 2.22 × 10^6^ CFU/μg plasmid at 80 bp (*p* < 0.001). When the homologous arm length was between 80–100 bp, the transformation efficiency plateaued, with an average of 2.29 × 10^6^ CFU/μg plasmid (Figure 1). When the homologous arm length was between 10–40 bp, the transformation efficiency of donor DNA without selection markers was 1–100 times higher than that of donor DNA with selection markers. As the homologous arm length increased, the difference decreased. When the homologous arm length was between 50–70 bp, the transformation efficiency of donor DNA without selection markers was 12–70% higher than that of donor DNA with selection markers. As the homologous arm length increased, the difference decreased. When the homologous arm length was between 80–100 bp, there was no difference between the two (Figure 1). This may be because donor DNA fragments with selection markers are larger, and when the homologous arms are shorter, they are less likely to undergo recombination with the chromosome, leading to inefficient repair of Cas9-mediated DSBs and low cell viability. Therefore, longer homologous arms are preferable when using larger donor DNA fragments.

### 3.2. Impact of Donor DNA Homologous Arm Length on Gene Editing Efficiency

**Figure 2:**
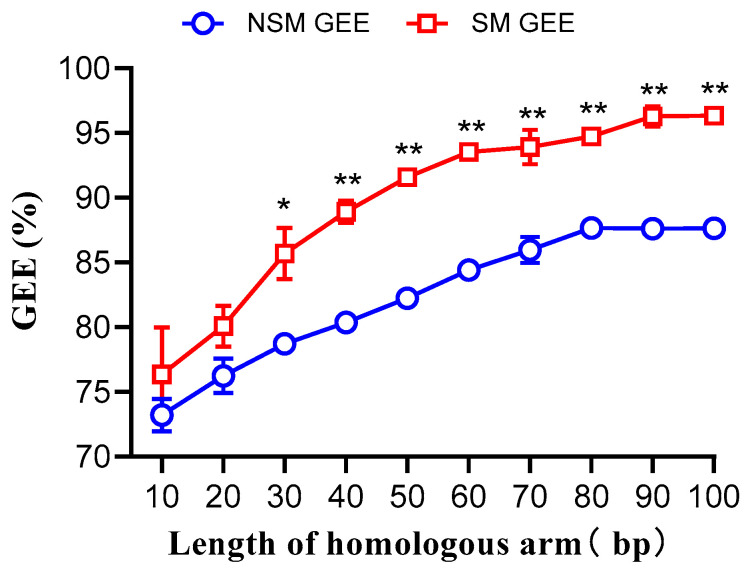
Gene editing efficiency (GEE) of donor DNA fragments with different lengths of homologous arms, with and without selection markers, co-transformed with pCas-SSB/pTargetF-*lacZ* into *Escherichia coli* MG1655. NSM, has not a selection marker. SM, selection marker. Data represent the mean of three independent experiments, with standard deviation (SD) as the error bars. Based on one-way ANOVA analysis. **, *p* < 0.001; *, *p* < 0.005.

Donor DNA fragments with varying lengths of homologous arms, both with and without selection markers, were co-transferred into MG1655 cells along with pCas-SSB/pTargetF-*lacZ* (Figure S5). For donor DNA fragments without selection markers, the gene editing efficiency increased linearly from 73% at 10 bp to 88% at 80 bp (*p* < 0.001). When the homologous arm length was between 80–100 bp, the gene editing efficiency reached a plateau, with an average of 88%. For donor DNA fragments with selection markers, the gene editing efficiency increased linearly from 76% at 10 bp to 93% at 60 bp (*p* < 0.001). When the homologous arm length was between 60–100 bp, the gene editing efficiency plateaued, with an average of 95%. There was no difference in gene editing efficiency between donor DNA fragments with and without selection markers when the homologous arm length was between 10–20 bp. However, when the homologous arm length was between 30–100 bp, the gene editing efficiency of donor DNA fragments with selection markers was 8–11% higher than that of donor DNA fragments without selection markers (Figure 2).

### 3.3. Impact of Donor DNA Homologous Arm Length on Homologous Recombination Rate

**Figure 3:**
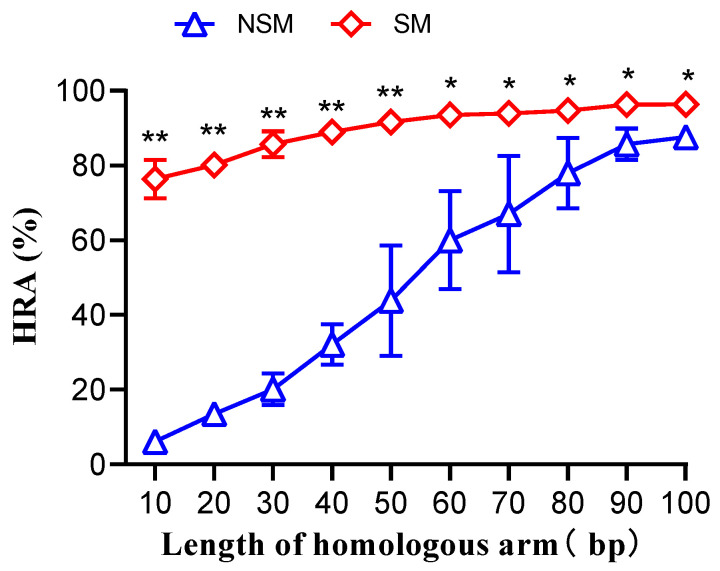
Homologous recombination rate (HRA) of donor DNA fragments with different lengths of homologous arms, with and without selection markers, co-transformed with pCas-SSB/pTargetF-*lacZ* into *Escherichia coli* MG1655. NSM, no selection marker; SM, selection marker. Data represent the mean of three independent experiments, with standard deviation (SD) as the error bars and based on one-way ANOVA analysis. **, *p* < 0.001; *, *p* < 0.005.

Donor DNA fragments with varying lengths of homologous arms, both with and without selection markers, were co-transformed into MG1655 cells along with pCas-SSB/pTargetF-*lacZ* (Figure S5). For donor DNA fragments without selection markers, the homologous recombination rate increased linearly from 6% at 10 bp to 86% at 90 bp (*p* < 0.001). When the homologous arm length was between 90–100 bp, the homologous recombination rate reached a plateau, with an average of 86%. The pattern of change in homologous recombination rate for donor DNA fragments with selection markers was similar to their gene editing efficiency, as all randomly selected white colonies underwent homologous recombination. When the homologous arm length was between 10–50 bp, the homologous recombination rate of donor DNA fragments with selection markers was 1–11 times higher than that of donor DNA fragments without selection markers, and as the homologous arm length increased, the difference in homologous recombination rate decreased. When the homologous arm length was between 60–100 bp, the homologous recombination rate of donor DNA fragments with selection markers was 10–56% higher than that of donor DNA fragments without selection markers, and, as the homologous arm length increased, the difference in homologous recombination rate decreased (Figure 3). The reason for the significantly lower homologous recombination rate of donor DNA fragments without selection markers, compared with those with selection markers in which the homologous arms are shorter, is that, when the homologous arms are shorter, gene editing events with donor DNA fragments without selection markers primarily occur through non-homologous end joining (NHEJ), while, with donor DNA fragments with selection markers, regardless of the length of the homologous arms, all gene editing events occur through homologous recombination to repair double-strand breaks (DSBs).

### 3.4. Impact of Selection Marker on Dual-Site Gene Editing with Donor DNA

**Figure 4:**
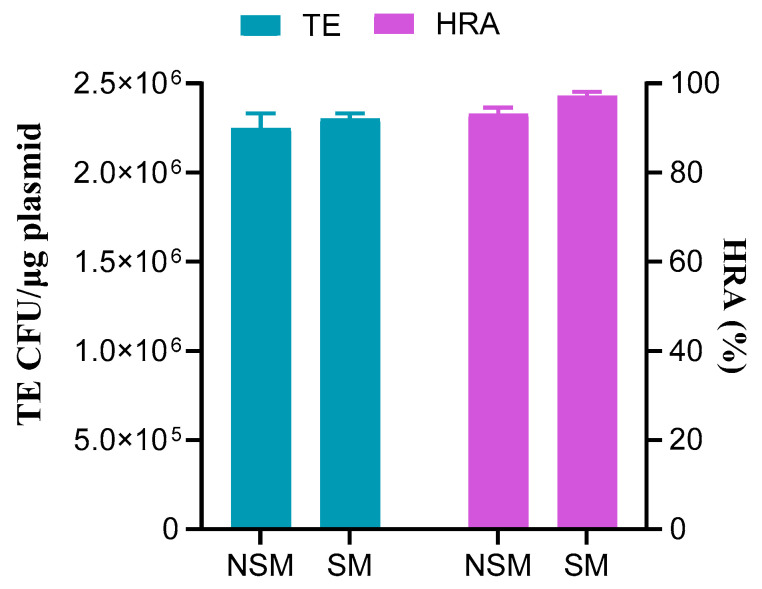
Transformation efficiency (TE) and homologous recombination rate (HRA) of donor DNA fragments with 100 bp homologous arms with and without selection marker for dual-site editing. NSM, no selection marker; SM, selection marker. Data represent the mean of three independent experiments, with standard deviation (SD) as the error bars.

Donor DNA fragments with and without selection markers, both having a homologous arm length of 100 bp, were separately co-transformed with pCas-SSB/pTargetF-*lacZ*-2 into MG1655 cells for dual-site editing of the *lacZ* gene (Figure S6). There were no differences in transformation efficiency or gene editing efficiency between donor DNA fragments with and without selection markers. Sequencing verification of selected white colonies confirmed homologous recombination-based gene editing occurred in all cases, indicating that the homologous recombination rate mirrored the gene editing efficiency. When both donor DNA fragments with and without selection markers simultaneously edited two sites on the *lacZ* gene, there were no differences in transformation efficiency or homologous recombination rate. Transformation efficiencies were both above 2.2 × 10^6^ CFU/μg plasmid, and homologous recombination rates were both above 93% (Figure 4). This suggests that the presence of a selection marker in donor DNA, or the total length of the donor fragment, does not affect dual-site editing when the homologous arm length is 100 bp.

### 3.5. Simultaneous Editing of Three Genes with 100 bp Homologous Arm Length Donor DNA Fragments without Selection Marker

**Figure 5:**
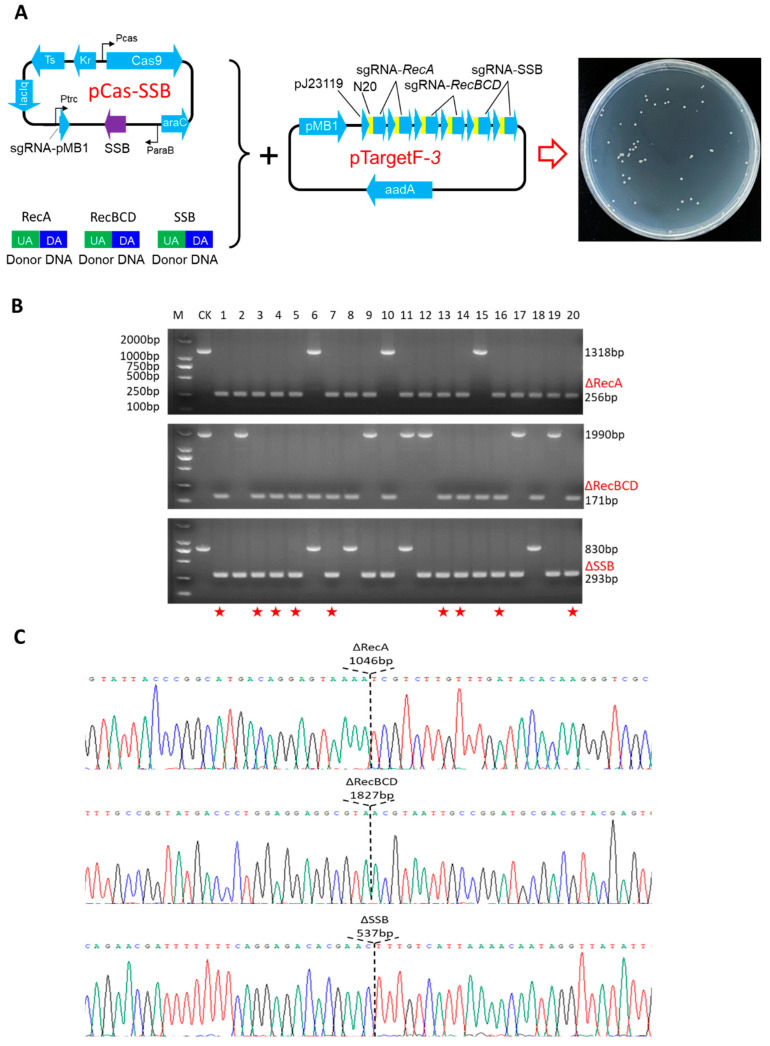
SSB/CRISPR-Cas9-mediated simultaneous knockout of three genes. (**A**) Schematic representation of transformation using three donor DNA fragments without antibiotic resistance expression cassettes. The pCas-SSB plasmid contains the streptococcus pyogenes Cas9 protein gene, driven by its native promoter P_cas_. The *Escherichia coli* SSB protein gene possesses an arabinose-inducible promoter P_araB_ and the arabinose-inducible transcription factor *araC*. The sgRNA-PMB1 is under the control of an IPTG-inducible promoter P_trc_ and guides the pMB1 replication, the elimination of pTargetF-3, lac repressor (lacIq), temperature-sensitive replication origin repA101 (Ts), and kanamycin resistance gene (Kr). Plasmid pTargetF-3 harbors two sgRNAs each targeting *RecA*, *RecBCD,* and *SSB* genes, directing two cleavage sites for each gene. (**B**) Gel electrophoresis image of transformants. The red pentagrams in the gel image represent transformants where all three genes have been simultaneously knocked out. (**C**) sequencing results of transformants with simultaneous knockout of three genes.

To achieve the simultaneous editing of three genes, plasmid pTargetF-3 was constructed. pTargetF-3 enables simultaneous expression of sgRNAs targeting N_20_ regions of *RecA*, *RecBCD*, and *SSB* genes. Additionally, linear dsDNA donor fragments targeting the deletion of *RecA*, *RecBCD*, and *SSB* genes via homologous recombination were separately constructed. These fragments lacked a selection marker and had a homologous arm length of 100 bp. The three donor dsDNA fragments were co-transformed with pCas-SSB/pTargetF-3 into MG1655 cells. Twenty randomly selected colonies were subjected to colony PCR, followed by sequencing analysis. The editing efficiency of individual genes was approximately 80%, with the probability of all three genes being simultaneously knocked out being approximately 45% (Figure 5).

## 4. Discussion

When using the CRISPR/Cas9 system for bacterial gene editing, the length of the homologous arms of dsDNA fragments typically range from 50 to 1000 bp [31,42,43], with considerable variation among different studies. Few studies have specifically focused on the optimal length of homologous arms. In this study, we investigated the efficiency of the SSB/CRISPR-Cas9 gene editing system for gene insertion and knockout in *Escherichia coli*, particularly focusing on the minimal homologous arm length required for effective gene editing. Our results demonstrate that, when using SSB/CRISPR-Cas9 for homologous recombination in *E. coli* with donor DNA fragments lacking selection markers, a homologous arm length of 50 bp achieves a gene editing efficiency of 82.22% and a homologous recombination rate of 53.33%. This is more than ten times higher than the efficiency reported by Sun et al. using the CT-CRISPR system with the same 50 bp homologous arm length [36]. Zhao et al. found that homologous arms shorter than 50 bp were ineffective for gene editing. By overexpressing the RecA and λ-Red recombination system, they were able to perform gene editing with a 41 bp homologous arm in *E. coli*, but the efficiency was only about 13.8%, requiring seven to eight colonies to obtain a successful edit [44]. 

In contrast, the SSB/CRISPR-Cas9 system, which substitutes SSB for the λ-Red recombination system, does not require RecA overexpression. Even with homologous arms shorter than 50 bp, the system achieves gene editing, with efficiencies exceeding 70% and a homologous recombination rate of approximately 5.55%, when the homologous arms are as short as 10 bp. This significantly reduces the difficulty and dependency when constructing homologous arms. An optimal homologous arm length of 80 bp yields a maximum gene editing efficiency of approximately 87.66% and a homologous recombination rate close to 100%. Due to the non-homologous recombination activity of the SSB protein, there can be instances of non-homologous edits among the transformants. When the homologous arm length reaches 100 bp, the homologous recombination rate can reach 100%. Therefore, when using the SSB/CRISPR-Cas9 system for homologous recombination in bacterial gene editing, the optimal length for donor DNA homologous arms is between 90 and 100 bp to achieve high editing efficiency. In special cases, homologous arms as short as 10 bp can still yield acceptable results.

When the donor DNA fragment contains a selection marker and antibiotics are added during the growth of transformants for screening, even with a homologous arm length as short as 20 bp, the gene editing efficiency can still exceed 80%. This is because the addition of antibiotics in the culture medium allows for the selection of transformants, thereby avoiding the growth of non-homologous recombination transformants. As the length of the homologous arms reaches 90 bp, the gene editing efficiency stabilizes, reaching 96.28%. That all of the transformants contain antibiotic resistance markers indicates successful homologous recombination. Hence, in utilizing the SSB/CRISPR-Cas9 system for bacterial gene editing with selection markers, the requirement for the length of the donor DNA homologous arms is not stringent. Homologous arms of 20 bp or more can generally meet the transformation requirements. Selection of donors with 60–100 bp homologous arms that contain selection markers can achieve efficient gene editing.

The CRISPR/Cas9 system commonly utilizes single target sites for bacterial gene editing [12,45], However, whether dual target sites can achieve better gene editing efficiency has not been specifically studied. When using the SSB/CRISPR-Cas9 system for gene editing, setting dual target sites for DNA double-strand breaks does not significantly affect the gene editing efficiency when compared with single target sites when the donor DNA fragment lacks selection markers. Hence, in practical applications, using a single target site can achieve high gene editing efficiency. However, when the donor DNA fragment contains selection markers, the gene editing efficiency achieved with dual target sites is significantly higher than that with a single target site (*p* < 0.01), reaching 97.29%. This is because dual target sites can induce longer segment deletions in the target region and can mitigate off-target effects associated with single target sites to some extent. Therefore, in practical applications where selection markers are required and the target gene to be knocked out is relatively long, dual target sites will provide higher gene editing efficiency.

The SSB/CRISPR-Cas9 system serves as a gene editing tool capable of simultaneously editing three genes, with editing efficiencies for individual genes exceeding 80%. Simultaneous editing of three genes achieves an efficiency of 45%, consistent with the findings of Jiang et al. [12]. Although this falls short of the 88.3% reported by Feng et al. [37], the process does not require the construction of donor fragments into vectors, thereby reducing operational complexity. The editing efficiency obtained is entirely sufficient for the construction of mutant strains. To our knowledge, there have been no reports of the simultaneous editing of three genes in bacteria using linear dsDNA as donor fragments. Utilizing the SSB/CRISPR-Cas9 gene editing system in conjunction with dsDNA offers a more convenient approach for multi-gene editing.

## Figures and Tables

**Table 1:** The bacterial strains and plasmids used in this study ^a^.

Strain or Plasmid	Description	Source or Reference
*E. coli*		
DH5α	F^−^ *endA1 glnV44 thi-1 recA1 relA1 gyrA96 deo*R *nup*G φ80d*lac*Z∆M15 ∆(*lac*ZYA-*arg*F) *U169 hsd*R17 (r_K_^−^ m_K_^+^) λ^−^	TaKaRa
MG1655	K-12; F^−^, λ^−^, *ilvG*^−^, *rfb*-50, *rph*-1	[40]
Plasmids		
pCas-SSB	*repA101*(Ts) *kan* P_cas_-*cas9* P_araB_-*SSB lacI*^q^ P_trc_-sgRNA-*pMB1*	[19]
pTargetF	*pMB1* aadA sgRNA	[12]
pTargetF-*lacZ*	*pMB1* aadA sgRNA-*lac*Z	[19]
pTargetF-*lacZ*-2	*pMB1* aadA sgRNA-*lacZ* sgRNA-*lac*Z2	This study
pTargetF-3	*pMB1* aadA sgRNA-*RecA*1 sgRNA-*RecA*2 sgRNA-*RecBCD*1 sgRNA-*RecBCD*2 sgRNA-*SSB*1 sgRNA-*SSB*2	This study

^a^ kan, kanamycin resistance gene; aadA, spectinomycin resistance gene; P_cas_-*cas9*, the cas9 gene with its native promoter; P_trc_-sgRNA-*pMB1*, sgRNA with an N_20_ sequence for targeting the *pMB1* region with a *trc* promoter; sgRNA-*lac*Z, sgRNA with an N20 sequence for targeting the *lac*Z locus; sgRNA-*lacZ*2, sgRNA with another N_20_ sequence for targeting the *lac*Z locus; sgRNA-*RecA*1, sgRNA with an N_20_ sequence for targeting the *RecA* locus; sgRNA-*RecA*2, sgRNA with another N_20_ sequence for targeting the *RecA* locus; sgRNA-*RecBCD*1, sgRNA with an N_20_ sequence for targeting the *RecBCD* locus; sgRNA-*RecBCD*2, sgRNA with another N_20_ sequence for targeting the *RecBCD* locus; sgRNA-*SSB*1, sgRNA with an N_20_ sequence for targeting the *SSB* locus; sgRNA-*SSB*2, sgRNA with another N_20_ sequence for targeting the *SSB* locus; *SSB*, the gene encoding the single-stranded DNA-binding protein of *E. coli*.

**Table 2:** The primers used in this study ^a^.

Primer	Sequence (5′-3′)
pC01	GGAAACAGCT AATTCTCATGTTTGA
pC02	CCGGTTATTA TATCTATATCGAGAT
pC03	ATTTCACACAGGAAACAGCT AATTCTCATGTTTGA
pC04	CATGGCCTGCCCGGTTATTA TATCTATATC GAGAT
pC05	GCGGATAACAATTTCACACAGGAAACAGCT AATTCTCATGTTTGA
pC06	TACGGGCAGACATGGCCTGCCCGGTTATTA TATCTATATC GAGAT
pC07	GGAATTGTGAGCGGATAACAATTTCACACAGGAAACAGCT AATTCTCATGTTTGA
pC08	TTACGCGAAATACGGGCAGACATGGCCTGCCCGGTTATTA TATCTATATC GAGAT
pC09	TATGTTGTGTGGAATTGTGAGCGGATAACAATTTCACACAGGAAACAGCT AATTCTCATGTTTGA
pC10	ATGGATTTCCTTACGCGAAATACGGGCAGACATGGCCTGCCCGGTTATTA TATCTATATC GAGAT
pC11	TTCCGGCTCGTATGTTGTGTGGAATTGTGAGCGGATAACAATTTCACACAGGAAACAGCT AATTCTCATGTTTGA
pC12	ATAGTACATAATGGATTTCCTTACGCGAAATACGGGCAGACATGGCCTGCCCGGTTATTA TATCTATATCGAGAT
pC13	CACTTTATGCTTCCGGCTCGTATGTTGTGTGGAATTGTGAGCGGATAACAATTTCACACAGGAAACAGCT AATTCTCATGTTTGA
pC14	TGTTTTTTAAATAGTACATAATGGATTTCCTTACGCGAAATACGGGCAGACATGGCCTGCCCGGTTATTA TATCTATATCGAGAT
pC15	CCAGGCTTTACACTTTATGCTTCCGGCTCGTATGTTGTGTGGAATTGTGAGCGGATAACAATTTCACACAGGAAACAGCT AATTCTCATGTTTGA
pC16	CAAAAGTTTGTGTTTTTTAAATAGTACATAATGGATTTCCTTACGCGAAATACGGGCAGACATGGCCTGC CCGGTTATTA TATCTATATCGAGAT
pC17	ATTAGGCACCCCAGGCTTTACACTTTATGCTTCCGGCTCGTATGTTGTGTGGAATTGTGAGCGGATAACAATTTCACACAGGAAACAGCT AATTCTCATGTTTGA
pC18	ACCGAACATCCAAAAGTTTGTGTTTTTTAAATAGTACATAATGGATTTCCTTACGCGAAATACGGGCAGA CATGGCCTGC CCGGTTATTA TATCTATATCGAGAT
pC19	TAGCTCACTCATTAGGCACCCCAGGCTTTACACTTTATGCTTCCGGCTCGTATGTTGTGTGGAATTGTGAGCGGATAACAATTTCACACAGGAAACAGCT AATTCTCATGTTTGA
pC20	AAAAGAATAAACCGAACATCCAAAAGTTTGTGTTTTTTAAATAGTACATAATGGATTTCCTTACGCGAAATACGGGCAGACATGGCCTGCCCGGTTATTA TATCTATATCGAGAT
pC21	CGGTAGTGGGATACGACGAT
pC22	CGGTTGGAATAATAGCGAGA
pC23	TCGTCAGGCTACTGCGTAT
pC24	ATCCAACAGGCGAGCAT
pC25	CGCAACAAGGGATTTACAC
pC26	GATGGCGGATTTAGGGT
pC27	GAACCGAGGTCACAACATA
pC28	TCACAATGGCGGACAT

^a^ The green and blue letter sequences correspond with the upstream and downstream homologous regions of the *lacZ* gene, while the yellow letter region is used to amplify the tetracycline resistance gene expression cassette.

## Data Availability

Data are contained within the article and Supplementary Materials.

## Supplementary Materials

The following supporting information can be downloaded at: https://www.mdpi.com/article/10.3390/microorganisms12061102/s1, Table S1: Synthesized Fragments of Different Lengths of Homologous Arms for Upstream and Downstream of the LacZ Gene Without Selection Markers; Figure S1: Synthesized Fragment for Constructing the pTargetF-lacZ-2 Plasmid; Figure S2: Synthesized Fragment for Constructing the pTargetF-3 Plasmid; Figure S3: Expression Cassette for the Tetracycline Resistance Gene; Figure S4: Donor DNA Fragments for Three Genes Without Selection Markers; Figure S5: Transformation of MG1655 with Homologous Arms of Different Lengths, with and without Selection Markers, Mediated by SSB/CRISPR-Cas9; Figure S6: Dual Target Site Editing of Target Genes with Two Different Homologous Fragments under SSB/CRISPR-Cas9 Mediation.
