# Supplementary material for: The Influence of Homologous Arm Length on Homologous Recombination Gene Editing Efficiency Mediated by SSB/CRISPR-Cas9 in Escherichia coli
Source: Microorganisms. 2024 May 29;12(6):1102. doi: 10.3390/microorganisms12061102 (PMC11205466; doi:10.3390/microorganisms12061102)
Supplement: Supplementary file 1 [file microorganisms-12-01102-s001.zip › microorganisms-3041396-supplementary.pdf]

**Table S1.** Synthesized Fragments of Different Lengths of Homologous Arms for Upstream and Downstream of the *LacZ* Gene Without Selection Markers*

| Homology arm length | Sequence (5’ -3’) |
|---|---|
| 10bp | GGAAACAGCTTAATAACCGG |
| 20bp | ATTTCACACAGGAAACAGCTTAATAACCGGGCAGGCCATG |
| 30bp | GCGGATAACAATTTCACACAGGAAACAGCTTAATAACCGGGCAGGCCATGTCTG<br>CCCGTA |
| 40bp | GGAATTGTGAGCGGATAACAATTTCACACAGGAAACAGCTTAATAACCGGGCAG<br>GCCATGTCTGCCCGTATTTCGCGTAA |
| 50bp | TATGTTGTGTGGAATTGTGAGCGGATAACAATTTCACACAGGAAACAGCTTAATA<br>ACCGGGCAGGCCATGTCTGCCCGTATTTCGCGTAAGGAAATCCAT |
| 60bp | TTCCGGCTCGTATGTTGTGTGGAATTGTGAGCGGATAACAATTTCACACAGGAA<br>ACAGCTTAATAACCGGGCAGGCCATGTCTGCCCGTATTTCGCGTAAGGAAATCC<br>ATTATGTACTAT |
| 70bp | CACTTTATGCTTCCGGCTCGTATGTTGTGTGGAATTGTGAGCGGATAACAATTTC<br>ACACAGGAAACAGCTTAATAACCGGGCAGGCCATGTCTGCCCGTATTTCGCGTA<br>AGGAAATCCATTATGTACTATTTAAAAAACA |
| 80bp | CCAGGCTTTACACTTTATGCTTCCGGCTCGTATGTTGTGTGGAATTGTGAGCGGA<br>TAACAATTTCACACAGGAAACAGCTTAATAACCGGGCAGGCCATGTCTGCCCGT<br>ATTTCGCGTAAGGAAATCCATTATGTACTATTTAAAAAACACAAACTTTTG |
| 90bp | ATTAGGCACCCCAGGCTTTACACTTTATGCTTCCGGCTCGTATGTTGTGTGGAATT<br>GTGAGCGGATAACAATTTCACACAGGAAACAGCTTAATAACCGGGCAGGCCATG<br>TCTGCCCGTATTTCGCGTAAGGAAATCCATTATGTACTATTTAAAAAACACAAAC<br>TTTTGGATGTTCGGT |
| 100bp | TAGCTCACTCATTAGGCACCCCAGGCTTTACACTTTATGCTTCCGGCTCGTATGTT<br>GTGTGGAATTGTGAGCGGATAACAATTTCACACAGGAAACAGCTTAATAACCGG<br>GCAGGCCATGTCTGCCCGTATTTCGCGTAAGGAAATCCATTATGTACTATTTAAAA<br>AACACAAACTTTTGGATGTTCGGTTTATTCTTTT |

*The green and blue regions represent the upstream and downstream regions, respectively.

CGGGATCCTTGACAGCTAGCTCAGTCCTAGGTATAATACTAGTCAATCCGCCGTTTGTTCCCAGTTTTAGAGCTAGAAATAGCAAGTTAAAATAAGGCTAGTCCGTTATCAACTTGAAAAAGTGGCACCGAGTCGGTGCtttttttccgaaaaaaaaaccccgcccctgacagggcggggttttttttaattaacagaaaattattttaaatttcctcTTGACAGCTAGCTCAGTCCTAGGTATAATACTAGTCGCCTGGCAGCAGTGGCGTCGTTTTAGAGCTAGAAATAGCAAGTTAAAATAAGGCTAGTCCGTTATCAACTTGAAAAAGTGGCACCGAGTCGGTGCTTTTTTTGAATTCCG

**Figure S1**. Synthesized Fragment for Constructing the pTargetF-*lacZ*-2 Plasmid
The fragment is 364 bp in length, with underlined regions indicating the BamHI and EcoRI restriction enzyme cutting sites. The yellow region represents the $N_{20}$ region, while lowercase letters denote the connecting regions between sgRNAs.

CGGGATCCTTGACAGCTAGCTCAGTCCTAGGTATAATACTAGTGGCCAGATTGAGAAAC
AATTGTTTTAGAGCTAGAAATAGCAAGTTAAAATAAGGCTAGTCCGTTATCAACTTGAA
AAAGTGGCACCGAGTCGGTGCtttttttccgaaaaaaaaaccccgcccctgacagggcggggttttttttaattaacagaaa
attattttaaatttcctcTTGACAGCTAGCTCAGTCCTAGGTATAATACTAGTCGACATCCGTCGTAT
CGGCGGTTTTAGAGCTAGAAATAGCAAGTTAAAATAAGGCTAGTCCGTTATCAACTTGA
AAAAGTGGCACCGAGTCGGTGCtttttttccgaaaaaaaaaccccgcccctgacagggcggggttttttttaattaacagaa
aattattttaaatttcctcTTGACAGCTAGCTCAGTCCTAGGTATAATACTAGTCCCACGCCGATGAT
CCTCTGGTTTTAGAGCTAGAAATAGCAAGTTAAAATAAGGCTAGTCCGTTATCAACTTG
AAAAAGTGGCACCGAGTCGGTGCtttttttccgaaaaaaaaaccccgcccctgacagggcggggttttttttaattaacag
aaaattattttaaatttcctcTTGACAGCTAGCTCAGTCCTAGGTATAATACTAGTTACTGAGCGGCGC
AGTGGTCGTTTTAGAGCTAGAAATAGCAAGTTAAAATAAGGCTAGTCCGTTATCAACTT
GAAAAAGTGGCACCGAGTCGGTGCtttttttccgaaaaaaaaaccccgcccctgacagggcggggttttttttaattaac
agaaaattattttaaatttcctcTTGACAGCTAGCTCAGTCCTAGGTATAATACTAGTGAAGTACGCTA
CATGCCAAAGTTTTAGAGCTAGAAATAGCAAGTTAAAATAAGGCTAGTCCGTTATCAAC
TTGAAAAAGTGGCACCGAGTCGGTGCtttttttccgaaaaaaaaaccccgcccctgacagggcggggttttttttaatta
acagaaaattattttaaatttcctcTTGACAGCTAGCTCAGTCCTAGGTATAATACTAGTCAGCCTCAGC
AGCCGCAGGGGTTTTAGAGCTAGAAATAGCAAGTTAAAATAAGGCTAGTCCGTTATCA
ACTTGAAAAAGTGGCACCGAGTCGGTGCTTTTTTTGAATTCCG

**Figure S2.** Synthesized Fragment for Constructing the pTargetF-3 Plasmid

The fragment is 1204 bp in length, with underlined regions indicating the BamHI and EcoRI restriction enzyme cutting sites. The green region represents the $N_{20}$ region, while lowercase letters denote the connecting regions between sgRNAs.

AATTCTCATGTTTGACAGCTTATCATCGATAAGCTTTAATGCGGTAGTTTATCACAGTTA
AATTGCTAACGCAGTCAGGCACCGTGTATGAAATCTAACAATGCGCTCATCGTCATCCT
CGGCACCGTCACCCTGGATGCTGTAGGCATAGGCTTGGTTATGCCGGTACTGCCGGGCC
TCTTGCGGGATATCGTCCATTCCGACAGCATCGCCAGTCACTATGGCGTGCTGCTAGCG
CTATATGCGTTGATGCAATTTCTATGCGCACCCGTTCTCGGAGCACTGTCCGACCGCTTT
GGCCGCCGCCCAGTCCTGCTCGCTTCGCTACTTGGAGCCACTATCGACTACGCGATCAT
GGCGACCACACCCGTCCTGTGGATCCTCTACGCCGGACGCATCGTGGCCGGCATCACC
GGCGCCACAGGTGCGGTTGCTGGCGCCTATATCGCCGACATCACCGATGGGGAAGATC
GGGCTCGCCACTTCGGGCTCATGAGCGCTTGTTTCGGCGTGGGTATGGTGGCAGGCCC
CGTGGCCGGGGGACTGTTGGGCGCCATCTCCTTGCATGCACCATTCCTTGCGGCGGCG
GTGCTCAACGGCCTCAACCTACTACTGGGCTGCTTCCTAATGCAGGAGTCGCATAAGG
GAGAGCGTCGACCGATGCCCTTGAGAGCCTTCAACCCAGTCAGCTCCTTCCGGTGGGC
GCGGGGCATGACTATCGTCGCCGCACTTATGACTGTCTTCTTTATCATGCAACTCGTAG
GACAGGTGCCGGCAGCGCTCTGGGTCATTTTCGGCGAGGACCGCTTTCGCTGGAGCGC
GACGATGATCGGCCTGTCGCTTGCGGTATTCGGAATCTTGCACGCCCTCGCTCAAGCCT
TCGTCACTGGTCCCGCCACCAAACGTTTCGGCGAGAAGCAGGCCATTATCGCCGGCAT
GGCGGCCGACGCGCTGGGCTACGTCTTGCTGGCGTTCGCGACGCGAGGCTGGATGGC
CTTCCCCATTATGATTCTTCTCGCTTCCGGCGGCATCGGGATGCCCGCGTTGCAGGCCAT
GCTGTCCAGGCAGGTAGATGACGACCATCAGGGACAGCTTCAAGGATCGCTCGCGGCT
CTTACCAGCCTAACTTCGATCATTGGACCGCTGATCGTCACGGCGATTTATGCCGCCTC
GGCGAGCACATGGAACGGGTTGGCATGGATTGTAGGCGCCGCCCTATACCTTGTCTGC
CTCCCCGCGTTGCGTCGCGGTGCATGGAGCCGGGCCACCTCGACCTGAATGGAAGCCG
GCGGCACCTCGCTAACGGATTCACCACTCCAAGAATTGGAGCCAATCAATTCTTGCGG
AGAACTGTGAATGCGCAAACCAACCCTTGGCAGAACATATCCATCGCGTCCGCCATCT
CCAGCAGCCGCACGCGGCGCATCTCGATATAGATA

**Figure S3.** Expression Cassette for the Tetracycline Resistance Gene

Each upstream and downstream homologous arm for the *RecA* gene is 100 bp.
CAATTTCTACAAAACACTTGATACTGTATGAGCATACAGTATAATTGCTTCAACAGAAC
ATATTGACTATCCGGTATTACCCGGCATGACAGGAGTAAAATCGTCTTGTTTGATACACA
AGGGTCGCATCTGCGGCCCTTTTGCTTTTTTAAGTTGTAAGGATATGCCATGACAGAAT
CAACATCCCGTCGCCCGGCATA

Each upstream and downstream homologous arm for the *RecBCD* gene is 100 bp.
AAGAACATCCGCAACAAGGGATTTACACAACCCGACCCAACGCCGGGTTGATTGCCCT
GATGGATGAGATGTTTGCCGGTATGACCCTGGAGGAGGCGTAACGTAATTGCCGGATG
CGACGTACGAGTGTTACGCATGTCGCATCCGACGATTTTCATCGCTTACCCTAAATCCG
CCATCAACACTTTGGATTTACGCTG

Each upstream and downstream homologous arm for the *SSB* gene is 100 bp.
TTCCCGGAACCGAGGTCACAACATAGTAAAAGCGCTATTGGTAATGGTACAATCGCGC
GTTTACACTTATTCAGAACGATTTTTTTCAGGAGACACGAACTTTGTCATTAAAACAAT
AGGTTATATTGTTTTAAGGTGGATGATTAAAGCATCTGCCAGCCATAAAAAAGAAGCCT
CCGTTATGGAGGCTTCTACGTATC

**Figure S4.** Donor DNA Fragments for Three Genes Without Selection Markers

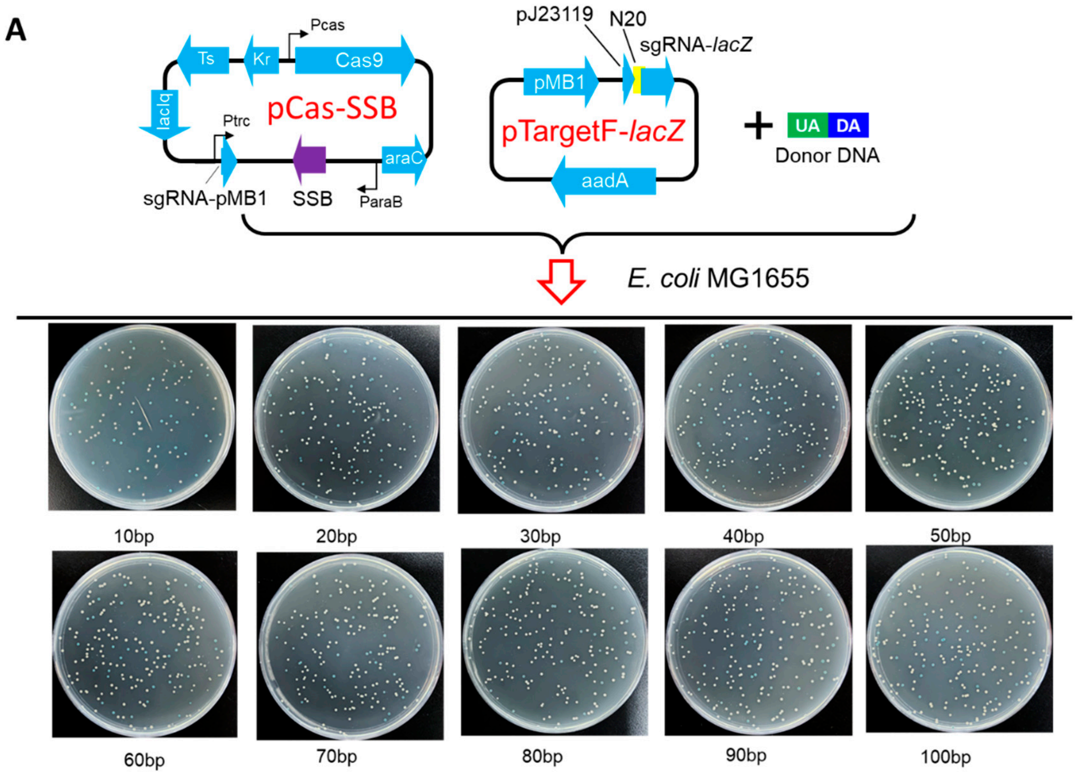


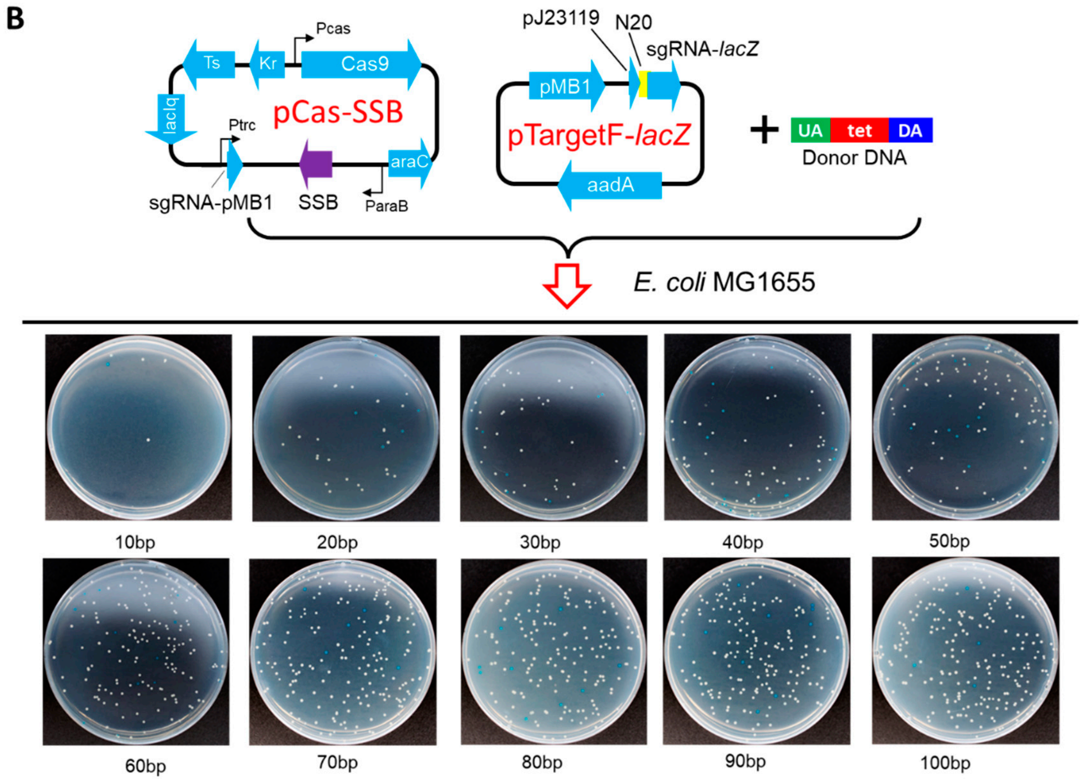


**Figure S5.** Transformation of MG1655 with Homologous Arms of Different Lengths, with and without Selection Markers, Mediated by SSB/CRISPR-Cas9

(A) Transformation results of homologous arm fragments of different lengths without selection markers under SSB/CRISPR-Cas9 mediation. (B) Transformation results of homologous arm

fragments of different lengths with selection markers under SSB/CRISPR-Cas9 mediation. The pCas-SSB plasmid contains the Streptococcus pyogenes Cas9 protein gene, driven by its native promoter $P_{cas}$. The *Escherichia coli* SSB protein gene possesses an arabinose-inducible promoter $P_{araB}$ and the arabinose-inducible transcription factor *araC*. The sgRNA-PMB1 is under the control of an IPTG-inducible promoter $P_{trc}$ and guides the pMB1 replication, elimination of pTargetF-*lacZ*, lac repressor (lacIq), temperature-sensitive replication origin repA101 (Ts), and kanamycin resistance gene (Kr). Plasmid pTargetF-3 harbors two sgRNAs each targeting *lacZ* gene.

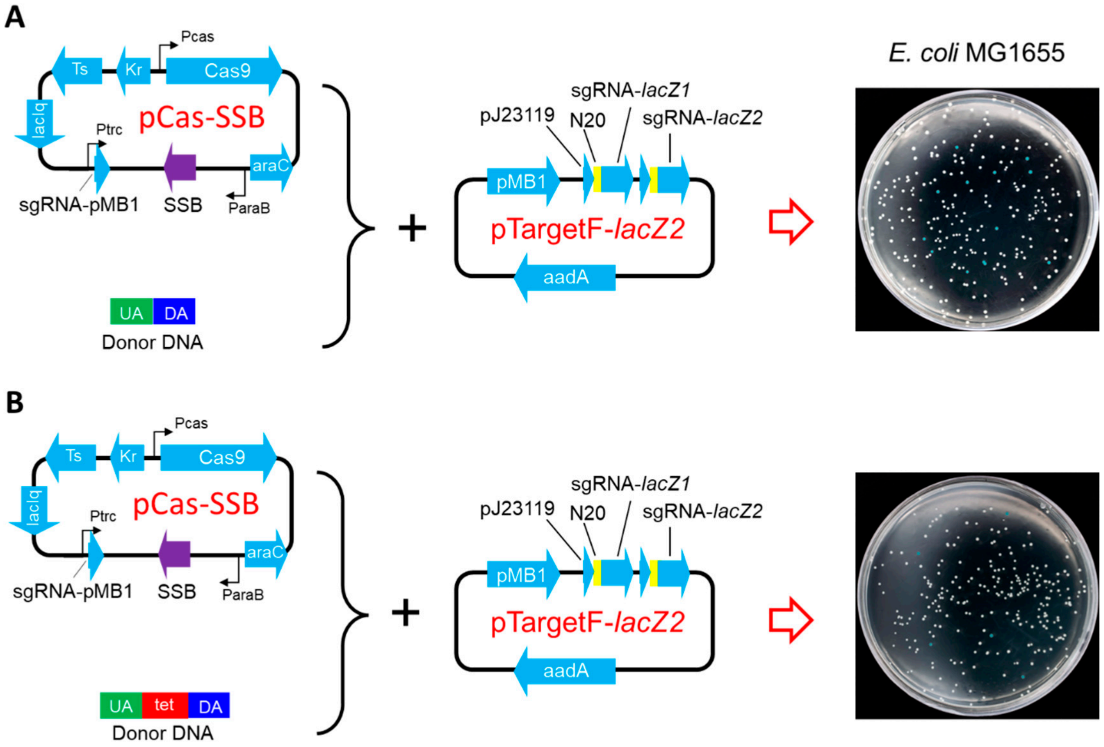


**Figure S6.** Dual Target Site Editing of Target Genes with Two Different Homologous Fragments under SSB/CRISPR-Cas9 Mediation

(A) Dual editing of the *lacZ* gene using homologous fragments without selection markers. (B) Dual editing of the *lacZ* gene using homologous fragments with selection markers. Plasmid pTargetF-*lacZ*-2 harbors two sgRNAs each targeting *lacZ* gene, directing two cleavage sites for *lacZ* gene.
